# Energy and nutrient intake increased by 47–67% when amylase was added to fortified blended foods—a study among 12‐ to 35‐month‐old Burkinabe children

**DOI:** 10.1111/mcn.12459

**Published:** 2017-05-03

**Authors:** Nynke A. Kampstra, Nguyen Van Hoan, Damiet J.P.C. Koenders, Rotraut Schoop, Britt C. Broersen, Claire Mouquet‐Rivier, Tahirou Traoré, Maaike J. Bruins, Saskia de Pee

**Affiliations:** ^1^ World Food Programme Rome Italy; ^2^ DSM Biotechnology Center Delft The Netherlands; ^3^ DSM Nutritional Products Kaiseraugst Switzerland; ^4^ Institut de Recherche pour le Développement Montpellier France; ^5^ Professionnels du développement solidaire Ouagadougou Burkina Faso

**Keywords:** amylase, complementary feeding, energy intake, fortified blended foods, infant and child nutrition, Super Cereal, Super Cereal Plus

## Abstract

Adding amylase to fortified blended foods can improve energy density, and increase child's energy and nutrient intake. The efficacy of this strategy is unknown for the World Food Programme's Super Cereal Plus (SC+) and Super Cereal (SC) blends. The primary goal of this study was to investigate the increased energy intake from amylase‐containing SC+ and SC compared to control porridges in Burkinabe children. Secondly, energy intake from amylase‐containing porridges compared to CERELAC^®^, Vitazom, and eeZeeBAR™ was studied. Thirdly, caregivers' (*n* = 100) porridge acceptability was investigated. The design was a randomized double‐blind controlled cross‐over trial studying the effect of amylase addition to SC+ and SC flours on porridge energy and nutrient intake in healthy Burkinabe children aged 12–23 (*n* = 80) and 24–35 months (*n* = 40). Amylase added to porridges increased energy density from 0.68 to 1.16 kcal/g for SC+ and from 0.66 to 1.03 kcal/g for SC porridges. Among children aged 12–23 months, mean energy intake from all porridges with amylase (135–164 kcal/meal) was significantly higher compared to control SC+ porridges (84–98 kcal/meal; model‐based average). Among children aged 24–35 months, mean energy intakes were also significantly higher from all porridges with amylase added (245–288 kcal/meal) compared to control SC porridges (175–183 kcal/meal). Acceptability of the porridges among caregivers was rated neutral to good, both for amylase‐added and non‐amylase‐containing porridges. These findings suggest that, among 12–35‐month‐old, adding amylase to fortified blended foods significantly increased energy and consequently nutrient intake per meal by 67% for SC+ and 47% for SC. Moreover, amylase‐containing porridges were well accepted by the caregivers.

## 1 INTRODUCTION

Adequate nutrition is essential for optimal child growth and development (Dewey, 2003). The first 1,000 days of life, from preconception to the end of the second year of life, are recognized as the most critical period (Black et al., 2013). To prevent undernutrition from the age of 6 months, energy and nutrient‐dense complementary foods are required in addition to breastfeeding in order to meet young children's recommended nutrient intakes (de Pee, 2015; Dewey, 2003; WHO, 2012).

The World Food Programme has developed two types of fortified blended foods, Super Cereal Plus (SC+) and Super Cereal (SC). SC+ contains corn/wheat/rice (52–58%), soy (20–25%), sugar (9%), milk powder (8%), oil (3%) and a vitamin and mineral premix, is produced by extrusion cooking, and is specifically used for the treatment of moderate acute malnutrition in children under 5 years old and for complementary feeding of children aged 6–23 months. SC is very similar to the traditional fortified blended foods (based on local staples, such as rice, wheat and corn), previously known as corn soy blend (CSB+), wheat soy blend (WSB+), or rice soy blend (RSB+). It contains corn/wheat/rice (57–64%), soy (24–30%) and a vitamin and mineral premix, and sugar may be included (10–11%). SC is provided to target groups other than children under five, such as pregnant and lactating women, or households that receive a food basket with a limited micronutrient content.

The energy density of 4 kcal/g dry matter recommended by the FAO/WHO (2013) guidelines on formulated complementary foods for older infants and young children is met by SC+ and SC flour. However, the average energy density of prepared SC+ and SC porridges at room temperature (earlier unpublished assessment found 0.70 and 0.56 kcal/g, respectively) is lower than that recommended by the WHO technical note on foods for moderate acute malnutrition treatment (WHO, 2012) and by the FAO/WHO in the Codex Standard for processed cereal‐based foods for infants and young children (FAO/WHO, 2006). Both guidelines state that energy density of cereal‐based foods for these target groups, that is, of SC+ in particular, should not be less than 0.8 kcal/g in prepared porridge.

Adding amylase to flour can reduce porridge viscosity and hence increase energy and nutrient density at a desired viscosity level (i.e., more dry matter can be included in the same volume of porridge without increasing viscosity). This can result in higher energy and nutrient intake in young children, which is important for meeting their nutrient requirements (FAO/WHO, 2013). Several studies have shown that the consumption of foods that include amylase increased young children's energy intake (Bennett et al., 1999; Hossain, Wahed, & Ahmed, 2005; Moursi, Mbemba, & Trèche, 2003; Van Hoan, Mouquet‐Rivier, Eymard‐Duvernay, & Treche, 2010; Van Hoan, Van Phu, Salvignol, Berger, & Trèche, 2009; Phu et al., 2012). Furthermore, two trials studying the effect on breast milk intake concluded that when breastfed children consumed energy‐dense foods containing amylase, breast milk intake was not affected (Moursi et al., 2003; Owino et al., 2007).

While these studies show the potential of adding amylase to foods to improve energy and nutrient intake among young children, the acceptability of SC+ and SC with amylase and the effects on energy and nutrient intake has not yet been investigated. The study aimed to test the hypothesis that SC+ and SC porridges with amylase were comparable, that is, non‐inferior, to SC+ and SC porridges without amylase. The primary objective was to compare energy intake per serving from SC+ and SC porridges with and without amylase, among 12‐ to 23‐and 24‐ to 35‐month‐old healthy Burkinabe children (consumption study). Energy intake from SC+ and SC with and without amylase was also compared to two locally available porridges, that is, CERELAC^®^ (instant) and Vitazom (locally produced), and to eeZeeBAR™ (instant). The secondary objective was to test porridge acceptability among their caregivers (sensory study).

**Key messages**

- Addition of amylase significantly increased energy intake and consequently nutrient intake per serving; by 67% for SC+ porridges in children aged 12–23 months and 47% for SC porridges in children aged 24–35 months.
- Caregivers' perceptions of amylase‐containing porridges were neutral to positive, and some porridges were significantly better rated than the non‐amylase‐added counterparts.
- Our findings indicate that children aged 12–23 months had a lower preference for porridges with higher sugar content.

## 2 METHODS

### 2.1 Study site

The study sites were located at three different health centers (in districts 1, 7, and 9) in rural Fada N'Gourma, province of Gourma, Burkina Faso. The consumption study was implemented between the 14 of June and the 13 of July 2014; the sensory study took place at the 15 of July. GRET's study team consisted of supervisors (*n* = 3), investigators (*n* = 6), and cooks (*n* = 30). The team was trained during 4 days on the study objectives, porridge preparation and distribution, data collection, and data recording. A medical doctor trained the team on dealing with possible adverse events and reactions such as choking in children.

### 2.2 Study population

**Figure 1:**
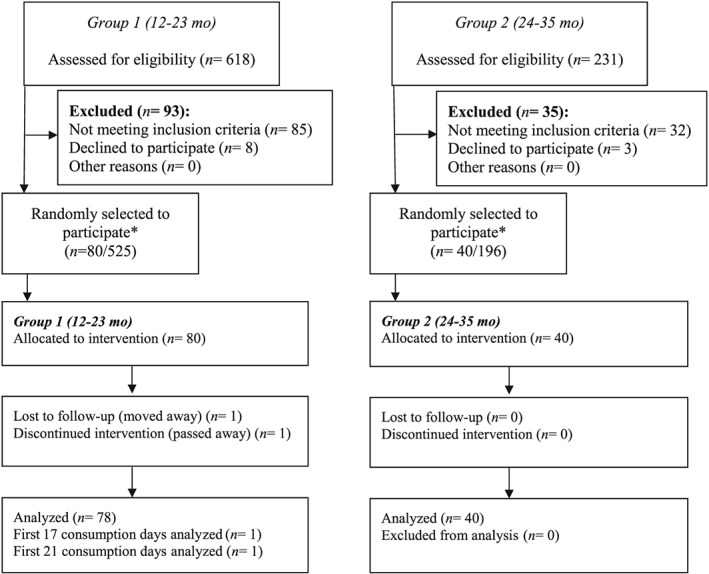
Flowchart for analysis sample. *Based on their proximity to health centers and agreement or commitment of the parents to participate for the complete duration study (30 consecutive days)

Caregivers of children aged 12–35 months were recruited by the study team in May 2014 (Figure 1). The study population was recruited using purposive and snowball sampling. Caregivers with children who were likely to fulfill the inclusion criteria and who were willing to participate were verbally informed about the study aims and procedures. If they consented to participate, the child's height, weight, and mid‐upper arm circumference (MUAC) were measured and medical history was assessed using a questionnaire. Their child was considered eligible when the following inclusion criteria were met: (a) 12–35‐month‐old, (b) MUAC > 115 mm, (c) used to consuming complementary foods in addition to breastfeeding (receiving breastfeeding itself was not considered as an inclusion criteria), (d) free from any medical complications and acute or chronic illness at recruitment, and (e) not enrolled in any feeding program. Caregivers' fingerprint signed consent forms were obtained when their child was eligible for study enrollment. The study population was divided into two groups: group 1 consisted of 80 children aged 12–23 months and group 2 of 40 children aged 24–35 months. The children in group 1 were assigned to health centers in districts 7 and 9 and group 2 to the health center in district 1. The study was performed in line with the ethical principles of the Declaration of Helsinki, as formulated by the World Medical Association and was approved by the Ministry of Health in Ouagadougou, Burkina Faso (WMA, 2013).

### 2.3 Sample size calculations

Sample size calculations were based on porridge intake per meal and its variation as observed among children of comparable age in two similar acceptability studies (unpublished observations). For children aged 12–23 months, porridge intake was estimated to be 38–45 g/meal with a within‐child standard deviation (SD, calculated on log transformed volume data) of 0.38–0.41. For children aged 24–35 months, porridge intake varied between 105 and 216 g/meal with a within‐child SD (calculated on log transformed volume data) of 0.24–0.28. For children aged 12–23 months, the sample size required to test the one‐sided non‐inferiority hypothesis with a non‐inferiority margin of 25%, at a 5% level of significance and with a power of 80%, was 66 to 73 children. For children aged 24–35 months, the necessary sample size was 25 to 35 children. Assuming a drop‐out rate of 10%, the final sample size was determined as 80 children aged 12–23 months and 40 children aged 24–35 months. Children aged 6–11 months were not included in the study as they do have a constant and stable enough consumption pattern yet, due to which sample size and study duration would have had to be larger. Sample size calculations were performed with the statistical software R, version 2.15.2 using the formula (6.19) described by Julious (“Sample Sizes for Clinical Trials”, CRC Press, 2010) for non‐inferiority cross‐over trials.

### 2.4 Flours studied

The SC+ and SC flours were based on four different types of cereal blends with and without amylase: CSB, WSB, and RSB, all produced by extrusion cooking (Michiels Fabrieken NV, Zulte, Belgium) and CSB drum dried. The SC WSB was not available due to production issues. SC+ and SC flours with and without amylase based on drum‐dried CSB were produced by Indofoods, Jakarta, Indonesia. The vitamin and mineral premixes used for these SC+ and SC flours, with and without α‐amylase (BAN 800MG, Novozymes, Copenhagen, Denmark) were produced by DSM Nutritional Products, Isando, South Africa. The α‐amylase was added in the same concentration for flours containing amylase to the vitamin–mineral premix for SC+ and SC flour by Michiels Fabrieken NV. SC+ and SC flours were packed in multi‐layered (PE/MET/Polyester) bags. Nutrient content, microbiology, heavy metal and toxin content of the SC+, and SC flours were analyzed by SGS laboratory in Hamburg, Germany.

In addition, three other infant cereal products were tested: eeZeeBAR™ (provided by GC Rieber, Compact, Søfteland, Norway), a wheat‐based compressed cereal bar that is crumbled and mixed with water to make a porridge, corn‐based CERELAC^®^ “my first maize cereal” (Nestlé product, sold in Burkina Faso), which is an instant porridge, and Vitazom (based on a blend of corn, soy, cowpea, and groundnut and containing α‐amylase), which was developed and produced by GRET, replaced SC WSB which was not available due to production issues. Vitazom and CERELAC^®^ were procured from the local distributer in Ouagadougou, Burkina Faso. The analysis report for eeZeeBAR™ sample was provided by GC Rieber. The nutritional composition of eeZeeBAR™, CERELAC^®^, and Vitazom was derived from the product label. Since the producers of eeZeeBAR™, CERELAC^®^, and Vitazom have to ensure that their products comply with prevailing quality, safety, and labeling regulations, it was not considered necessary to double‐check the products' declared macronutrient and micronutrient profiles.

### 2.5 Consumption study

The double‐blind cross‐over trial was implemented over a period of 30 consecutive days during which caregivers were asked to feed their child at the study site where porridge intake of the child was measured. The caregivers were advised not to feed the child, including breast milk, for at least 1 hr before the study site visit in the early morning. In group 1 (12–23 months), children consumed the following 10 porridges: SC+ CSB, WSB, RSB and drum‐dried CSB, each with and without amylase, eeZeeBAR™, and CERELAC^®^. In group 2 (24–35 months), children consumed the following 10 porridges: SC CSB, RSB, and drum‐dried CSB, each with and without amylase, WSB with amylase, Vitazom with amylase, eeZeeBAR™, and CERELAC^®^. The 10 different porridges were randomly assigned to the children in a cross‐over design, that is, in a different order. Each child received one porridge for three consecutive days. The sequence of porridge consumption was determined using FIZZ Acquisition software (Biosystèmes, Couternon, France). There were 10 different sequences, and per group, eight (group 1) or four (group 2) children were fed porridges in the same sequence.

Prior to the study's implementation, porridges were prepared during a 4‐day training period to determine the appropriate water/flour ratio, and the porridges were pretested in order to determine the locally preferred consistency, which could finally be described as a spoonable consistency, able to make a heap on a spoon. This was done under supervision of a food technologist from GRET and experts from DSM and World Food Programme, together with the supervisors of the three selected study sites. Bags of flour were stored under dry and hygienic conditions. In order to blind the cooks and participants to the type and brand of product, all SC+ and SC flours were packed in identical, number‐coded bags, the CERELAC^®^ can was wrapped and number‐coded, the outer packaging of the eeZeeBAR™ was removed, and a number code was added. Vitazom flour was packaged in a transparent plastic bag. The coded bags were transported to the respective sites on a daily basis. In order to meet the recommended fat content, 4 g oil/100 g flour was added to the seven SC porridges (group 2) in the field.

Data collection forms and record sheets were translated into French by an official translator. In order to prepare each porridge type, a pot, spoon, gas cooker, a weighing scale, and stopwatch were used by the cooks at the study sites. All equipments were coded to ensure that the same equipment was used for preparing the same porridge every day. The cooks received porridge‐specific preparation instructions and prepared the same porridge once every day in the morning around 5 a.m., for 30 days. Precise weight of flour and water (and oil for the SC porridges) used as well as final porridge weight were recorded daily by the cooks and was used to calculate porridge dry matter content (i.e., by water loss during cooking). Prepared porridges were stored in thermos flasks to keep it warm and for easy distribution.

The supervisors poured approximately 200 g (group 1) to 250 g (group 2) of porridge into a coded bowl. They were responsible for weighing the bowl when handing out the porridge and when the caregiver returned the bowl. On each study site, every child had their own subject number (ranging from 1 to 40 or from 1 to 80), corresponding to the porridge sequence they had been assigned to. Flasks and bowls were coded with the porridge number. The caregivers were asked to spoon‐feed the porridge to their child ad libitum. When the child showed signs of having had enough to eat, the caregivers were instructed to retry feeding a couple of times. Estimated porridge losses were then subtracted from the difference between the total weight of the bowl and porridge before consumption and after consumption, to determine the amount of porridge consumed more precisely. In addition, caregivers were given clean preweighed napkins to collect the spilt porridge. All weighing was done using an electronic scale with 0.1 g precision (Furi ML‐CF1). Samples of each prepared porridge were collected to determine dry matter content by dehydration in an oven at 105 °C until constant weight. Each day, porridge flow rate was measured using the Bostwick consistometer at 45 °C following the standard procedures as earlier described (Mouquet, Greffeuille, & Treche, 2006). Bostwick flow rate is expressed in millimeter per 30 s and can range from 0 (almost solid porridge) to 240 mm/30 s (very fluid porridge).

### 2.6 Sensory study

Caregivers' acceptability and preference for the different porridges were studied 2 days after completion of the 30‐day consumption study. The caregivers' acceptability of the different porridges was assessed by a sensory test. All caregivers (*n* = 120) were invited to taste five different porridges to perform a quantitative sensory and ranking test.

The caregivers received five porridges in a randomized sequence as generated by the program FIZZ Acquisition. The porridges were prepared using the same procedure as used for the 30‐day consumption study. The investigators carried out the sensory test in a face‐to‐face setting with the caregivers. Caregivers were asked to evaluate the porridges for (a) taste, (b) smell, (c) color, (d) consistency, and (e) overall sensory impression. Responses were rated by using a 5‐point hedonic scale, that is, 1 (very bad), 2 (bad), 3 (neutral), 4 (good), and 5 (very good) that was supported by corresponding faces in pictogram style representing satisfaction or dissatisfaction with the product (Msaddak et al., 2015; Murray, Delahunty, & Baxter, 2001).

### 2.7 Energy and nutrient intake calculations

Intake per serving and dry matter content after porridge cooking were used to derive energy intake. Mean energy density was 0.66 kcal/g for SC+ and 0.68 kcal/g for SC porridges without amylase (Table 2). This was 1.16 kcal/g for amylase‐containing SC+ and 1.03 kcal/g for amylase‐containing SC porridges. While for SC+, both sugar (9% by kcal) and oil (3% by kcal) are part of the product's specification and added in the factory; their addition to SC is optional. In the present study, sugar (9% by kcal) was added to the SC flour in the factory, while oil (4 g oil/100 g flour) was added during porridge preparation. The presented energy density (kcal/100 g) accounts for sugar and oil content. Macronutrient and micronutrient contents per 100 g flour analyzed by laboratory of Société Générale de Surveillance (SGS, Hamburg, Germany) were used to calculate total protein (g), fat (g), vitamin A (μg RE), iron (mg), calcium (mg), and potassium (mg). In case of Vitazom, eeZeeBAR™, and CERELAC^®^, the data on the label were used for calculations.

### 2.8 Statistical analysis

#### 2.8.1 Analysis for the consumption study

For each age group, a separate intention‐to‐treat analysis was performed. The study protocol and statistical analysis plan specified a linear mixed model with child as random factor and with fixed effects sex, day rank of diet period (days 1, 2, or 3), order of food distribution (period in which the porridge was served), and porridge type. Age in months was included as an additional explanatory covariate.

The following possible additional confounders, some of which were not known at the writing of the statistical analysis plan, were investigated by including each of them as additional fixed effect and performing a likelihood ratio test versus the original model for the main outcomes “energy intake in kilocalorie per meal” and “porridge consumption in grams per meal”: canteen (for the younger group only), surveyor (person handing out and weighing the bowls), and sequence (order of porridge consumption). Since “sequence” turned out to be significant for the group with children aged 24–35 months, the final model for this group included sequence as additional fixed effect.

Model assumptions were checked visually by evaluating goodness of fit plots. Energy and nutrient intake data of the children aged 12–23 months were square root transformed to adjust for skewness and backtransformed for their interpretation. Thus, average of model‐based means for porridge groups was backtransformed into original units, using the statistical software R. Energy and nutrient intake are thus presented as means and 95% confidence intervals, instead of means ± SD. Adjustments for multiple comparisons using Tukey contrasts were carried out to test significant mean differences (Hothorn, Bretz, & Westfall, 2008). Levels of significance were based on these adjusted *p*‐values. Data were analyzed with the statistical software R version 3.0.2 (R Core Team, 2013), applying the add‐on packages nlme version 3.1‐117 (Pinheiro et al., 2014), multcomp version 1.3‐6 (Hothorn et al., 2008), and lsmeans version 2.11 (Lenth, 2014).

#### 2.8.2 Sensory study

Quantitative data of the sensory study were entered in Epidata version 3.1 (The EpiData Association, Odense, Denmark). The porridge sensory rankings regarding taste, smell, color, consistency, and overall ranking were analyzed with analysis of variance. Adjustments for multiple comparisons using a Bonferroni correction were carried out to test for significant mean differences between porridges. Pearson's chi‐square test was used to evaluate data available on porridge ranking, using a significance level of *p* < .05. Data were analyzed using SAS software 9 for Windows (SAS Institute Inc., Cary, NC, USA) and Statgraphics Centurion XVI software (version 16.1.18; Statpoint Technologies Inc., Warrenton, VG, USA).

## 3 RESULTS

In total, 120 eligible children with their caregivers were enrolled in the study, that is, 80 in group 1 with children aged 12–23 months and 40 in group 2 with children aged 24–35 months. After consumption day 17, one child from group 1 was lost to follow‐up since the family moved away from the area, and on day 21, a child from group 1 passed away due to malaria. Consumption data of these two children were incomplete; however, all available data were used for analysis. In total, 100 of the caregivers of children who had participated in the consumption trial were enrolled for participation in the sensory study, that is, a response rate of 83% (*n* = 73 from group 1 and *n* = 27 from group 2).

### 3.1 Consumption study

#### 3.1.1 Characteristics of the children

Baseline characteristics of the children are presented in Table 1. In group 1, 47 boys and 33 girls were enrolled. In group 2, 24 boys and 16 girls were enrolled. Mean age was 17.8 ± 3.3 months in group 1 and 29.8 ± 4.0 months in group 2. Mean bodyweight (BW) was 8.8 ± 1.2 kg in group 1 and 11.3 ± 1.5 kg in group 2. Mean MUAC (mm) was 135 ± 9 for group 1 and 141 ± 10 for group 2.

**Table 1:** Baseline characteristics of children in group 1 (12–23 months) and group 2 (24–35 months)

Characteristics	Group 1 (*n* = 80)	Group 2 (*n* = 40)
Age (months), *Mean ± SD*	17.8 ± 3.3	29.8 ± 4.0
Sex, girls, % (*n*)	41.3 (33)	40.0 (16)
Breastfed, % (*n*)	73.7 (59)	2.6% (1)
Weight (kg), *Mean ± SD*	8.8 ± 1.2	11.3 ± 1.5
Minimum	6.1	7.5
Maximum	12.4	14.5
Height (cm), *Mean ± SD*	77.1 ± 4.3	87.8 ± 4.0
Minimum	65	79
Maximum	88	97
MUAC (mm), *Mean ± SD*	135 ± 9	141 ± 10
Minimum	120	123
Maximum	161	165
Child health status^a^, % (*n*)		
Well	87.3 (69)	81.6 (31)
Mild illness (without medical treatment)	3.8 (3)	2.6 (1)
Moderate illness (with medical treatment)	8.9 (7)	15.8 (6)
Adverse health symptoms^a^, % (*n*)		
Vomiting	2.6 (2)	5.2 (2)
Fever	10.1 (8)	13.1 (5)
Diarrhea	7.5 (6)	13.1 (5)
Difficulties breathing (e.g., a cold, coughing)	21.5 (17)	26.3 (10)
Skin rash	10.1 (8)	13.1 (5)
Lost appetite	10.1 (8)	21 (8)
Other symptoms	1.2 (1)	—

MUAC = mid‐upper arm circumference.

a Child health status and adverse health symptoms were recalled by the mothers.

#### 3.1.2 Porridge characteristics

Porridge's Bostwick flow rate, dry matter content after cooking, and energy density are presented in Table 2. As recommended in the publicly available technical specifications for the manufacture of SC+ and SC, the energy density should be 0.70 kcal/g for SC+ and 0.57 kcal/g for SC (without added sugar). In this study, mean energy density as measured in the field was 0.66 kcal/g for SC+ and 0.68 kcal/g for SC porridges. This increased to 1.16 and 1.03 kcal/g when amylase was added to SC+ and SC porridge, respectively (Table 2). Bostwick flow rate results for the eeZeeBAR™, CERELAC^®^, Vitazom, and SC drum‐dried CSB porridges were lower compared to the other prepared porridges, indicating thicker consistency. The variation of dry matter content of the porridges prepared over the different days was low for all porridges, indicating good reproducibility. The consistency measurements showed a higher variation, likely due to variation of porridge temperature in the field as has been described before (Mouquet et al., 2006). Differences in heating rate, which affects the time available for amylase to act on gelatinized starch, could play a role as well.

**Table 2:** Characteristics of the porridges consumed by group 1 (*n* = 80) and group 2 (*n* = 40)

Porridge	Bostwick flow rate (mm/30 s)	Dry matter content after cooking (g DM/100 g)	Energy density^a^ (kcal/100 g)
Group 1 (12–23 months)			
(1) SC+ CSB	194 ± 40	16.7 ± 1.3	71.3 ± 5.6
(2) SC+ CSB (amylase)	176 ± 22	26.6 ± 1.3	114.4 ± 5.6
(3) SC+ WSB	215 ± 25	17 ± 1.8	72.6 ± 7.5
(4) SC+ WSB (amylase)	201 ± 19	26.2 ± 1.8	113.1 ± 7.9
(5) SC+ RSB	205 ± 17	14.8 ± 0.6	63.9 ± 2.5
(6) SC+ RSB (amylase)	178 ± 26	27.1 ± 1.5	118.8 ± 6.4
(7) SC+ CSB drum drying	197 ± 31	13.5 ± 1.2	57.4 ± 5.3
(8) SC+ CSB drum drying (amylase)	123 ± 41	27.6 ± 1.4	118.8 ± 6.2
(9) eeZeeBAR™	126 ± 23	24.7 ± 0.4	114 ± 1.7
(10) CERELAC^®^	93 ± 15	25.0 ± 0.9	105.4 ± 3.8
Group 2 (24–35 months)			
(11) SC CSB	182 ± 33	14.8 ± 1.2	64.3 ± 5.3
(12) SC CSB (amylase)	226 ± 25	22.2 ± 0.6	96.7 ± 2.6
(13) Vitazom (amylase)	94 ± 28	22.6 ± 0.3	93 ± 1.1
(14) SC WSB (amylase)	191 ± 14	22.4 ± 0.3	96.5 ± 1.6
(15) SC RSB	166 ± 10	15.8 ± 1.1	69.3 ± 4.7
(16) SC RSB (amylase)	188 ± 15	23.6 ± 0.5	103.2 ± 2.1
(17) SC CSB drum drying	155 ± 19	15.7 ± 0.6	69.1 ± 2.7
(18) SC CSB drum drying (amylase)	92 ± 20	25 ± 0.9	109.4 ± 4.0
(19) eeZeeBAR™	112 ± 20	24.8 ± 0.5	114.4 ± 2.3
(20) CERELAC^®^	87 ± 18	24.9 ± 0.3	104.7 ± 1.2

All data are presented in means ± SD.

CSB = corn soy blend; DM = dry matter; RSB = rice soy blend; SC = Super Cereal; SC+ = Super Cereal Plus; WSB = wheat soy blend.

a Dry matter contents after cooking were used to determine porridge energy density and energy intake.

#### 3.1.3 Porridge consumption

**Figure 2:**
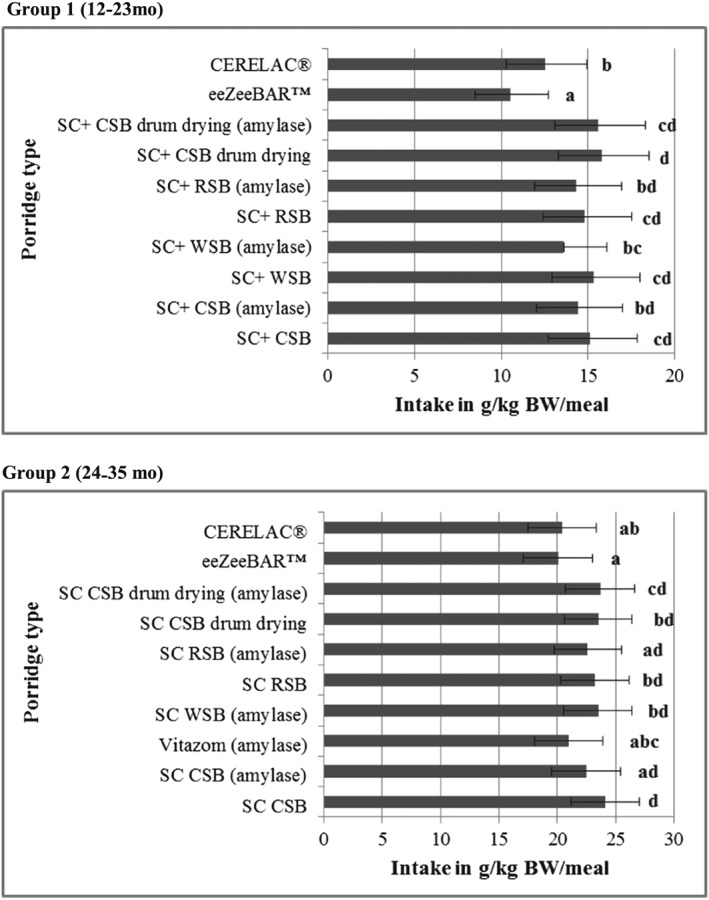
Meal size by bodyweight (in g/kg BW per meal). All data are presented in means and 95% confidence intervals. Values in a column with different superscript letters are significantly different, *p* < .05 (*p*‐values adjusted for multiple comparisons)

**Figure 3:**
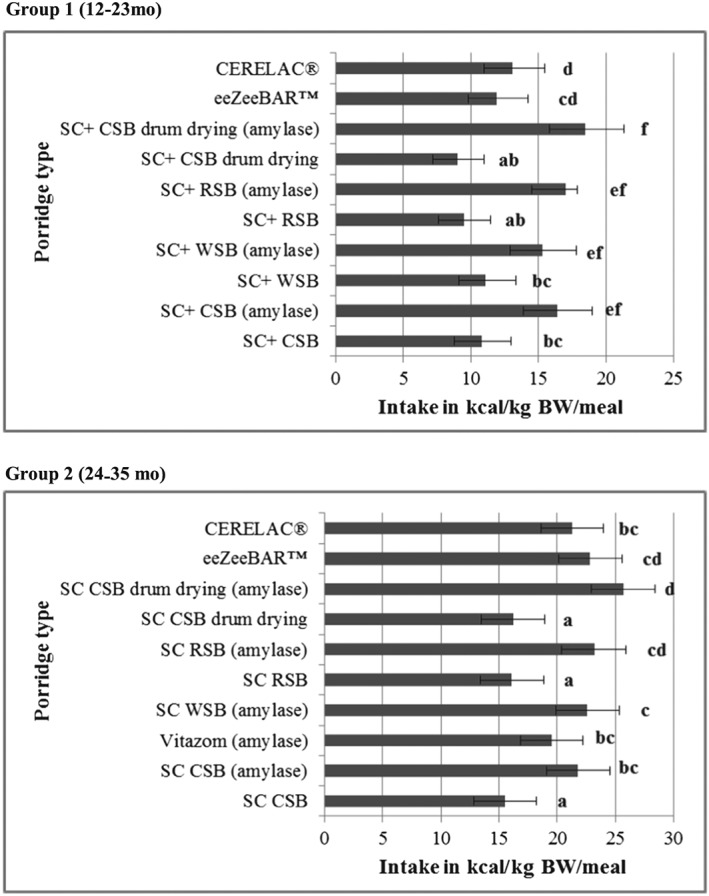
Energy intake per meal by bodyweight (in kcal/kg BW per meal). All data are presented in means and 95% confidence intervals. Values in a column with different superscript letters are significantly different, *p* < .05 (*p*‐values adjusted for multiple comparisons)

**Figure 4:**
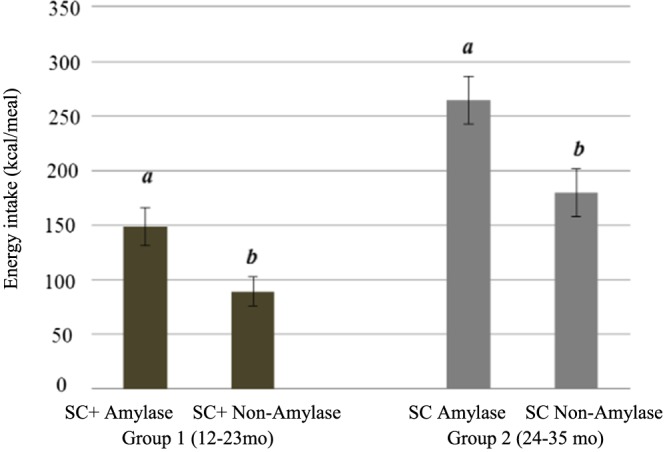
Model‐based mean porridge energy intakes (kcal/meal) from SC+ (12–23 months) and SC (24–35 months), with and without amylase. Data are presented in mean intake in kilocalorie per meal and 95% confidence intervals. Within SC+ and SC, bars with different letters are significantly different (i.e., energy intakes are significantly different between amylase and non‐amylase‐added porridges (*p* < .01). SC+ with and without added amylase includes CSB, WSB, RSB, and CSB drum dried. SC with and without amylase includes CSB, RSB, and CSB drum dried

The amount consumed in grams per kilogram BW per meal did not differ significantly between porridges both with and without amylase in both groups (Figure 2). In group 1, the model‐based mean ranged from 10.5 to 15.8 g/kg BW per meal. In group 2, this ranged from 20.1 to 24.1 g/kg BW per meal. In both age groups, energy intake (kcal/kg BW per meal) was significantly higher for each SC+ porridge with added amylase compared to their respective counterpart without amylase (*p* < .05; Figure 3). Overall, mean energy and conclusively the nutrient intake of the porridges with added amylase showed a 67% (SC+) and 47% (SC) increase compared to the porridges without amylase (*p* < .001; Figure 4). For age group 1, mean energy intake was significantly lower for the eeZeeBAR™ (106 kcal/meal) compared to all four SC+ porridges with amylase individually. Mean energy intakes were significantly lower for the CERELAC^®^ (11.9 kcal/kg BW per meal) porridge as compared to the SC+ porridges with added amylase, except for the SC+ WSB with amylase. For age group 2, CERELAC^®^ (21.3 kcal/kg BW per meal) and eeZeeBAR™ (22.8 kcal/kg BW per meal) showed similar energy intakes compared to the SC porridges with amylase (Figure 4).

### 3.2 Sensory study

Porridges that contained amylase were well accepted among caregivers (Table 3). With regard to the “overall” porridge scores, the acceptability appears somewhat higher for SC+ and SC porridges with amylase but was only significant for SC+ CSB drum dried and SC RSB porridges with amylase (Table 3). The instant vanilla flavored CERELAC^®^ porridge however consistently received the highest overall ratings for taste, smell, and overall ranking. In group 1, 71% and in group 2, 67% of the caregivers who evaluated the vanilla flavored CERELAC^®^ porridge ranked this as “liked most” (Table 4). In group 1, SC+ WSB and SC+ CSB drum dried, both without amylase, were ranked as “least liked” by 43% and 46% of the caregivers, respectively. In group 2, the caregivers ranked as “least liked” for SC CSB (50%) and SC CSB drum dried (60%), both without amylase.

**Table 3:** Overview of porridge sensory evaluation (mean ± *SD*) by caregivers of groups 1 (*n* = 73) and 2 (*n* = 27)

Porridge	Color	Consistency	Smell	Taste	Overall	Rank (based on the item overall)
Group 1 (12–23 months)						
(1) SC+ CSB	3.84 ± 0.14	3.49 ± 0.17	3.66 ± 0.16	2.74 ± 0.18	3.41 ± 0.13	8
(2) SC+ CSB (amylase)	3.17 ± 0.19	3.54 ± 0.17	3.69 ± 0.16	3.31 ± 0.21	3.59 ± 0.20	5
(3) SC+ WSB	3.19 ± 0.19	2.92 ± 0.19	3.08 ± 0.21	2.70 ± 0.21	2.94 ± 0.22	10
(4) SC+ WSB (amylase)	2.97 ± 0.19	3.00 ± 0.18	3.43 ± 0.18	3.57 ± 0.18	3.50 ± 0.20	6
(5) SC+ RSB	3.95 ± 0.15	3.71 ± 0.13	3.21 ± 0.1	2.63 ± 0.16	3.44 ± 0.17	7
(6) SC+ RSB (amylase)	3.86 ± 0.12	3.92 ± 0.16	3.36 ± 0.18	3.36 ± 0.15	3.94 ± 0.15	3
(7) SC+ CSB DD	4.09 ± 0.16	3.44 ± 0.17	2.83 ± 0.21	2.37 ± 0.24	3.03 ± 0.20	9
(8) SC+ CSB DD (amylase)	4.44 ± 0.09	4.28 ± 0.17	3.89 ± 0.20	4.08 ± 0.18	4.09 ± 0.17	2
(9) eeZeeBAR™	3.95 ± 0.14	3.22 ± 0.23	4.19 ± 0.14	4.43 ± 0.13	3.71 ± 0.15	4
(10) CERELAC^®^	4.32 ± 0.14	4.26 ± 0.15	4.66 ± 0.13	4.92 ± 0.06	4.62 ± 0.11	1
Group 2 (24–35 months)						
(11) SC CSB	3.08 ± 0.23	3.67 ± 0.23	2.92 ± 0.29	2.67 ± 0.28	3.25 ± 0.25	8
(12) SC CSB (amylase)	3.21 ± 0.21	3.21 ± 0.21	3.36 ± 0.13	3.29 ± 0.19	3.29 ± 0.19	7
(13) Vitazom (amylase)	2.38 ± 0.21	3.85 ± 0.34	4.38 ± 0.18	3.69 ± 0.21	3.23 ± 0.26	9
(14) SC WSB (amylase)	3.31 ± 0.26	3.77 ± 0.28	3.77 ± 0.23	4.00 ± 0.20	3.62 ± 0.24	4
(15) SC RSB	4.25 ± 0.18	4.25 ± 0.22	3.42 ± 0.19	2.67 ± 0.28	3.58 ± 0.26	5
(16) SC RSB (amylase)	4.18 ± 0.18	3.82 ± 0.23	3.82 ± 0.18	3.73 ± 0.20	3.82 ± 0.18	2
(17) SC CSB DD	4.73 ± 0.15	3.47 ± 0.34	3.07 ± 0.18	2.60 ± 0.27	3.07 ± 0.23	10
(18) SC CSB DD (amylase)	4.31 ± 0.21	3.46 ± 0.29	3.62 ± 0.21	3.62 ± 0.21	3.31 ± 0.26	6
(19) eeZeeBAR™	3.65 ± 0.19	3.29 ± 0.22	3.71 ± 0.17	4.35 ± 0.17	3.71 ± 0.21	3
(20) CERELAC^®^	4.00 ± 0.24	3.93 ± 0.23	4.60 ± 0.16	4.60 ± 0.16	4.67 ± 0.16	1

All values are presented as descriptive statistics in mean ± SD.

1 = very bad; 2 = bad; 3 = neutral, 4 = good; 5 = very good.

CSB = corn soy blend; DD = drum drying; SC = Super Cereal; SC+ = Super Cereal Plus; RSB = rice soy blend; WSB = wheat soy blend.

**Table 4:** Overview of the ranking test results with caregivers of children in group 1 (*n* = 73) and group 2 (*n* = 27)

Porridge	Caregivers scoring the porridge (*n*)	Liked most (*n*)	Second (*n*)	Third (*n*)	Fourth (*n*)	Least liked (*n*)
Group 1 (12–23 months)						
(1) SC+ CSB	38	0	7	11	11	9
(2) SC+ CSB (amylase)	35	5	10	6	6	8
(3) SC+ WSB	37	2	3	13	3	16
(4) SC+ WSB (amylase)	35	4	7	3	13	8
(5) SC+ RSB	38	5	4	9	9	11
(6) SC+ RSB (amylase)	36	5	12	10	6	3
(7) SC+ CSB DD	35	3	7	3	6	16
(8) SC+ CSB DD (amylase)	36	11	9	8	8	0
(9) eeZeeBAR™	37	10	8	8	8	3
(10) CERELAC^®^	38	27	7	2	2	0
Group 2 (24–35 months)						
(11) SC CSB	12	1	1	1	3	6
(12) SC CSB (amylase)	14	1	0	3	8	2
(13) Vitazom (amylase)	13	2	2	5	2	2
(14) SC WSB (amylase)	13	1	6	1	4	1
(15) SC RSB	12	2	5	1	2	2
(16) SC RSB (amylase)	11	2	3	2	3	1
(17) SC CSB DD	15	1	1	1	3	9
(18) SC CSB DD (amylase)	13	2	1	6	2	2
(19) eeZeeBAR™	17	5	5	6	0	1
(20) CERELAC^®^	15	10	3	1	1	0

CSB = corn soy blend; DD = drum dried; SC = Super Cereal; SC+ = Super Cereal Plus; RSB = rice soy blend; WSB = wheat soy blend.

## 4 DISCUSSION

The addition of amylase to the different SC+ and SC flour blends significantly increased energy density, and consequently nutrient intake proportionally, by 67% among children aged 12–23 months and 47% among children aged 24–35 months. Moreover, the amount of porridge consumed did not significantly change by adding amylase; hence, greater nutrient intakes are related to the increased energy density of the porridges, which is in contrast with previous findings that suggested a lower intake amount in grams per meal with higher energy‐dense amylase‐containing porridges (Bennett et al., 1999; den Besten, Glatthaar, & Ijsselmuiden, 1998; Sanchez‐Grinan, Peerson, & Brown, 1992; Vieu, Traoré, & Tréche, 2001). However, the effects of amylase on reducing the volume of intakes may only become visible after several days (Islam, Peerson, Ahmed, Dewey, & Brown, 2006).

The energy needs for children aged 12–23 months from complementary foods with “average” breast milk intake in developing countries is 550 kcal/day (Dewey, 2003). When consuming SC+ CSB without amylase (71.3 kcal/100 g, Table 2), the child would need to consume 770 ml porridge in order to meet this. If SC+ CSB with amylase is consumed (114.4 kcal/100 g, Table 2), the amount needed to meet a child's energy needs would be reduced to 480 ml/day to meet the recommended 550 kcal/day, about 38% less.

The children 12–23 months of age consumed 10.5–15.8 g/kg BW per meal, and the children 24–35 months of age consumed 20.1–24.1 g/kg BW per meal. This is less than the assumed functional gastric capacity of 30 g/kg BW for young children (which is 249 g/meal at 6–8 months, 285 g/meal at 9–11 months, and 345 g/meal at 12–23‐month‐old; Dewey & Brown, 2003; FAO/WHO, 2013). Similar low intakes per meal have also been reported by Mouquet‐Rivier, Traoré, Soma, Kaboré, and Trèche (2016); they showed that in 6‐ to 20‐month‐old Burkinabe children mean amounts of porridge consumed ranged from 7.6 to 19.7 g/kg BW per meal. Traoré, Vieu, Alfred, and Serge (2005) observed that Burkinabe infants aged 6–9 months consumed 6.3–9.0 g/kg BW. Van Hoan et al. (2009) reported a consumption of 14.2 and 15.3 g/kg BW among Vietnamese children aged 6–10 months in two experimental groups where amylase was added with significantly higher intakes for amylase‐containing foods compared to the homemade porridges. Another study by Van Hoan et al. (2010) among infants aged 6–10 months showed a mean porridge consumption of 11.8 to 17.7 g/kg BW per meal, that is, also lower than the assumed gastric capacity of 30 g/kg BW. Children only eat up to their maximum gastric capacity if the meal energy density is very low and energy intake from breast milk is very low as reported by Dewey and Brown (2003) and FAO/WHO (2013), which was not the case in the present study and may explain why breastfed children were consuming considerably less than their theoretical gastric capacity. The differences in porridge consumption by children as reported by different papers may be related to differences in study design, location, age of the study population, and the ingredients and consistency of the intervention products. External factors such as being fed in a different setting in combination with the large number of people on the sites could create a more distracting environment for the child, which may affect appetite and total energy intake per serving.

The intake of the various SC+ and SC porridges compared to two locally available porridges (CERELAC^®^ and Vitazom) and eeZeeBAR™ were different between the two age groups. In the younger children, eeZeeBAR™ (106 kcal/meal) and CERELAC^®^ (117 kcal/meal) porridge intakes were significantly lower compared with most porridges with amylase (model‐based mean 148 kcal/meal). In the older children, mean energy intake from CERELAC^®^ (242 kcal/meal) and eeZeeBAR™ (258 kcal/meal) were comparable to the porridges with amylase (model‐based mean 264 kcal/meal). The energy intake from Vitazom despite the addition of amylase, studied only in the older children (24–35 months), was relatively low (217 kcal/meal).

Although the energy intake from the eeZeeBAR™ porridge was comparable to that from SC porridges with amylase added, average meal intake (g/meal) for eeZeeBAR™ was significantly lower. It is likely that total energy intake is higher due to the higher fat content in eeZeeBAR™ (17%) compared to SC flour (6%). It should be noted that eeZeeBAR™ has 20% of carbohydrates coming from sugar, which is 12.6 g/100 g (12.6%). Sugar content for SC blends (10%) and SC+ blends (9%) was lower. CERELAC^®^ contains about 28% sugar, including lactose from its high skimmed milk powder content (~25% compared to ~8% in SC+), which markedly increases the sweetness of the product. Moreover, CERELAC^®^ porridge is flavored with vanilla which can improve its acceptability. Data on intake, however, do not show that the children liked CERELAC^®^ or eeZeeBAR™ better than SC porridge as they did not eat more (neither amount nor energy content) from these porridges. In fact, children aged 12–23 months consumed less of the porridges (in energy) with a higher sugar content (CERELAC^®^ and eeZeeBAR™) compared to the porridges with amylase. The older infants (24–35 months) did not have a preference towards porridges with a higher or lower sugar content. Vieu et al. (2001) found, in a study among 24 Burkinabe breastfed infants aged 6–10 months, that energy intake from porridges with sugar content of 9% and 20% did not significantly differ but was significantly higher compared to porridges with 1% sugar. Another study has reported higher preferences for sweeter porridges (Ventura & Mennella, 2011).

Several studies showed that adding amylase to flours in complementary foods is an effective strategy to increase energy intake in young children (Bennett et al., 1999; Moursi et al., 2003; Van Hoan et al., 2009; Van Hoan et al., 2010; Phu et al., 2012). Studied in younger children (4–8 months), Moursi et al. (2003) showed that children had a 52% higher median energy intake from porridges prepared with the amylase‐containing flour. One study showed however that there were no significant differences between the Chilenje baby mix with (106 kcal/100 g) or without (68 kcal/100 g) amylase in terms of total energy intake in 9‐month‐old Zambian infants (Owino et al., 2007). Furthermore, one of the studies showed that the addition of amylase was an effective strategy in malnourished children (Hossain et al., 2005), and another study showed this after recovery from diarrhea in a health care setting (Rahman et al., 1994). The children enrolled in this study were selected on the basis of having a good health status, that is, absence of any medical complication and having a MUAC > 115 mm. Results may therefore not be applicable to children with complications or severe acute malnutrition.

In the present study, breastfeeding rates were high among the study population at baseline. In group 1, 59 out of 80 mothers of the children aged 12–23 months breastfed their child. In group 2 with the children aged 24–25 months, one out of 40 mothers breastfed her child (Table 1). The caregivers were asked not to feed their child 1 hr before coming to the study site, but this was difficult to control. In case the children were breastfed before, it could have affected porridge intake negatively. This study did not monitor whether breastfeeding practices changed throughout the 30‐day study period, which can be seen as a limitation of the study design. However, as shown by other studies, breast milk consumption is not expected to be affected by adding amylase‐rich flours to the diets of children (Hossain et al., 2005; Moursi et al., 2003). Another limitation is that this study took place in well‐controlled ideal conditions and it was not studied using the effectiveness study design, also known as a real‐world study. Finally, we relied on the information provided by the caregivers regarding their child's health status, adverse health symptoms, and breast milk intake. Therefore, recall bias should be considered as a limitation.

Samples of each prepared porridge of all study sites were collected to determine porridge consistency by dry matter content and by Bostwick flow rate at 45 °C. The variation of the dry matter content as measured by dehydration was low for all porridges, indicating good reproducibility. It should be noted that after day 3 the water content for porridges 3 (SC+ WSB), 8 (SC+ CSB drum dried with amylase), and 18 (SC CSB drum‐dried amylase) was increased as the porridge consistency was found to be too thick, impacting the Bostwick flow rate measurements and its variation. It is known that porridge consistency depends on the preparation techniques in the field such as temperature (Mouquet et al., 2006). Although a thermometer was used to reach the right temperature for measuring every porridge Bostwick flow rate, the porridge consistency was variable, with the largest variability found for porridges 1 and 8.

Caregivers' perceptions of amylase‐containing porridges were neutral to positive, and some porridges were significantly better rated than the non‐amylase‐added counterparts. The sensory study among caregivers showed that all porridges on the organoleptic item “overall” were accepted, that is, above the predefined acceptability threshold of 3 (Neutral). Caregivers gave relatively high scores for the eeZeeBAR™ and CERELAC^®^ porridge on all organoleptic items. As discussed earlier, CERELAC^®^ claims about 28 g/100 g flour or 14 g per 50 g serving coming from sugars, has a high milk powder content, and is flavored with vanilla, which can positively impact its acceptability to the mothers. It has been reported before that adolescents and adults preferences are towards sweet sensations (Hayes & Duffy, 2008; Lesschaeve & Noble, 2005). For eeZeeBAR™, the technology (baking) and composition (oat, more fat, and sugar) may also have influenced its acceptability.

In conclusion, amylase addition increased the energy density of SC+ and SC porridges to 1.16 and 1.03 kcal/g, respectively. Addition of amylase significantly increased energy intake and consequently nutrient intake per serving; by 67% for SC+ porridges in children aged 12–23 months and 47% for SC porridges in children aged 24–35 months. The increased energy and nutrient intake of porridges with added amylase increases the likelihood that children meet their recommended nutrient intakes. High energy‐dense foods are recognized to be particularly important for meeting nutrient requirements in children of this study aged 12–35 months, but also for infants 6–12 months. Therefore, the possibilities of adding amylase, as a standard ingredient, to SC+ and SC should be further explored.

## CONFLICTS OF INTEREST

DK, RS, and MB are employed at DSM, a manufacturer of vitamin and mineral premixes. All other authors did declare no conflict of interest. The views expressed in this article are those of the listed authors.

## CONTRIBUTIONS

The authors' responsibilities were as follows: NVH, SdP, CMR, TT, DK, RS, and MB designed the study; NVH acted as study manager; NK monitored in the field; RS, TT, and NK performed the sample size calculations and statistical analyses; NK, BB, SdP, and NVH wrote the first draft of the manuscript; SdP had primary responsibility for the final content of the manuscript. All authors read and approved the final manuscript.
